# *Gut Pathogens*: enteric health at the interface of changing microbiology

**DOI:** 10.1186/1757-4749-1-1

**Published:** 2009-02-03

**Authors:** Niyaz Ahmed, Leonardo A Sechi, Francis Megraud, Seyed E Hasnain

**Affiliations:** 1 Pathogen Biology Laboratory, Department of Biotechnology, University of Hyderabad, Hyderabad, India; 2 Department of Biomedical Sciences, University of Sassari, Sassari, Italy; 3 INSERM – U853, Laboratoire de Bactériologie, CHU Pellegrin, 33076 Bordeaux cedex, France; 4 Institute of Life Sciences, Hyderabad University Campus, Hyderabad, India

As the open access movement begins revolutionizing the publication of publicly funded biomedical research, it is time to ensure access to important scientific discoveries and opinions within and across the disciplines which have direct bearing on health and quality of life in resource poor settings and in countries of the world where library budgets are seriously shrinking. In this concern, the area of enteric infectious diseases and gut health has been of paramount significance. For example, the enteric infections that cause diarrhoeal diseases already constitute one of the top 10 causes of death and kill approximately 1.81 million people in the developing world (6.9% of total deaths), a figure which is more dramatic and alarming than the number of deaths associated with tuberculosis and malaria put together (Source: The WHO) [1].

To this end, the International Society for Genomic and Evolutionary Microbiology (ISOGEM) in collaboration with BioMed Central Ltd. has launched *Gut Pathogens *with the aim of providing a high-quality forum for research on enteric infections of humans and animals. The journal led by three Editors-in-Chief and supported by a highly qualified and organized international Editorial Board [2] publishes open access research articles of repute in areas of biology and the pathogenesis of bacterial, parasitic and viral infections of the gut including their diagnosis, epidemiology and clinical management.

Besides publication of peer-reviewed research articles and case reports, *Gut Pathogens *is also mandated to publish scholarly reviews and other opinionated articles related to microbial etiology of gut ailments; microbial invasion mechanisms; bacterial toxins and virulence factors of the pathogens of gut and associated glands (liver, pancreas); bacterial adaptation and evolution of pathogenicity; molecular and serological diagnoses; treatment, vaccines and antimicrobial resistance mechanisms; molecular epidemiology, transmission dynamics and evolutionary genetics and comparative and veterinary infectious diseases of the gut.

The field of medical microbiology has expanded tremendously in the aftermath of genomic revolution [3], and microbial organisms in the gut have already taken center stage among all other microbiota of the human surfaces [4-6]. Metagenomics and systems biology approaches [4,5] have enabled new insights [6] into the complex existence of the gut ecosystem and hold tremendous potential to boost our understanding of the elements of host-microbial co-evolution, commensalism versus parasitic invasions and the boundaries thereof. In view of such an exciting and fast-moving scenario, there is a clear opportunity for *Gut Pathogens *to be seen also as a timely initiative to serve communities associated with a) biology and ecology of gut pathogens, the commensals and the probiotic species [7,8] in health and disease, b) host susceptibility or resistance to such organisms, c) immune reactions of gut infections and d) the triggers of autoimmunity in inflammatory bowel diseases [9,10] and other metabolic syndromes [11,12].

As like all BioMed Central journals, *Gut Pathogens *shall be committed to the open access publication policy – the concept that has revolutionized research access to communities and that was recently endorsed by major funding agencies including the NIH and the Wellcome Trust. In addition to its embracement of open access, *Gut Pathogens *shall also aim to support the concept of Science 2.0 [13] by providing a truly open platform for discussions, commenting and rating of the subjective impact of individual articles.

In the near future, medical and biosciences communities will need to possibly consider novel findings and paradigms that are emerging in the aftermath of genome sequencing and metagenomics of the gut microorganisms. Also, those who diagnose and treat patients with enteric disorders will need to understand the nature and complexities of the interactions of the so called 'invaders' [14] with our intestinal ecosystem in health, and their perturbations in disease, and the roles and contribution of our gut symbionts and commensals in guarding and ensuring normalcy of the gut or otherwise. This shall greatly facilitate the practice of preventive and social medicine in a most effective and pertinent manner. *Gut Pathogens *will always strive to assist scientists and clinicians in this endeavor by translating the most recent advances for the benefit of patients.
